# Biochanin A Induces Cuproptosis and Inhibits Lung Metastasis in triple-negative Breast Cancer

**DOI:** 10.32604/or.2026.084760

**Published:** 2026-10-28

**Authors:** Limei Zhang, Yue Duan, Shuang Pu, Pingting Chen, Xiaofang Xie, Yiming Liu, Zhaokai Zhou, Hailin Tang, Lei Yu, Danping Chen, Fu Peng

**Affiliations:** 1 West China School of Pharmacy, Sichuan University, Chengdu, China; 2 Department of Oncology, Chongqing Hospital of Traditional Chinese Medicine, Chongqing, China; 3 The First Clinical Medical College, Guangzhou University of Chinese Medicine, Guangzhou, China; 4 State Key Laboratory of Southwestern Chinese Medicine Resources, Chengdu University of Traditional Chinese Medicine, Chengdu, China; 5 Graduate School of Pharmaceutical Sciences, Kyoto University, Kyoto, Japan; 6 Department of Urology, The Second Xiangya Hospital of Central South University, Changsha, China; 7 State Key Laboratory of Oncology in South China, Guangdong Provincial Clinical Research Center for Cancer, Sun Yat-Sen University Cancer Center, Guangzhou, China; 8 Department of Pharmacology, Key Laboratory of Drug-Targeting and Drug Delivery System of the Education Ministry, Sichuan Engineering Laboratory for Plant-Sourced Drug and Sichuan Research Center for Drug Precision Industrial Technology, Sichuan University, Chengdu, China

**Keywords:** Biochanin A, TNBC, cuproptosis, lung metastasis, LIPT1

## Abstract

**Background:** Triple-negative breast cancer (TNBC) constitutes an invasive breast tumor subtype, marked by scarce available therapies and unfavorable patient prognosis. Natural products have attracted increasing attention as potential anticancer agents due to their multitarget activities and relatively low toxicity. This study aimed to investigate the antitumor effects of Biochanin A (BCA) in TNBC and to explore its underlying molecular mechanisms. **Methods:** The effects of BCA on TNBC cell proliferation, migration, and invasion were evaluated *in vitro*. Transcriptomic profiling, network pharmacology analysis, and mechanistic experiments were conducted to investigate the potential involvement of cuproptosis. The antimetastatic effects of BCA were further assessed *in vivo* using a mouse model. **Results:** BCA significantly inhibited the proliferative, migratory, and invasive capacities of TNBC cells. Integrative bioinformatics analyses identified cuproptosis as a potential mechanism underlying its antitumor activity. Mechanistic investigations suggested that BCA induces cuproptosis, accompanied by dysregulation of copper homeostasis, increased oxidative stress, mitochondrial dysfunction, and altered expression of cuproptosis-related proteins. Bioinformatics analysis further suggested that LIPT1 may be involved in this process. *In vivo* experiments demonstrated that BCA markedly suppressed lung metastasis in TNBC mouse models. **Conclusions:** BCA exhibits potent antitumor and antimetastatic activities against TNBC, potentially through the induction of cuproptosis, with LIPT1 possibly involved in this process. These findings provide experimental evidence supporting further investigation of BCA as a promising therapeutic candidate for TNBC.

## 1 Introduction

TNBC is a distinct subtype of breast cancer characterized by the absence of estrogen receptor (ER), progesterone receptor (PR), and human epidermal growth factor receptor 2 (HER2) [1,2]. Breast cancer remains the most frequently diagnosed malignancy among women worldwide, with approximately 2.3 million new cases and over 660,000 deaths reported in 2022 [3]. TNBC accounts for approximately 10%–20% of all breast cancer cases globally. Epidemiological studies have revealed marked geographic and racial disparities in TNBC incidence. The highest absolute burden is observed in Asia, followed by Europe and North America. TNBC is more frequently diagnosed in younger and premenopausal women, particularly in populations from low- and middle-income countries. Notably, the incidence of TNBC among women of African ancestry is nearly twice that observed in White women, suggesting that both genetic susceptibility and socioeconomic factors contribute to its development and progression [4,5]. Among breast cancer subtypes, TNBC is associated with a particularly poor prognosis and high mortality rate. Owing to the lack of well-defined molecular targets, current treatment strategies largely rely on conventional chemotherapy [6]. Clinically, TNBC is characterized by aggressive behavior, pronounced biological heterogeneity, early recurrence, and a strong tendency toward distant metastasis, all of which contribute to an unfavorable clinical outcome [7]. Recent studies have further highlighted substantial molecular and clinical heterogeneity within TNBC [8]. In particular, differences between HER2-low and HER2-negative subgroups within TNBC-like populations have been reported, including variations in metabolic activity, immune cell infiltration, and tumor microenvironmental features, which may influence both tumor aggressiveness and therapeutic responsiveness [9]. Although emerging therapeutic strategies, including immune checkpoint inhibitors and antibody–drug conjugates, have improved outcomes in selected patients with TNBC, significant clinical challenges remain. Immunotherapy targeting the PD-1/PD-L1 axis has demonstrated encouraging efficacy in a subset of patients, particularly in those with PD-L1-positive tumors [10,11]. However, the overall response rate remains limited due to heterogeneity in PD-L1 expression, variability in tumor mutational burden (TMB), and the presence of intrinsic or acquired resistance mechanisms [12,13]. Consequently, only a fraction of patients derive durable clinical benefit from current immunotherapeutic approaches. Despite recent advances in systemic therapies, the overall prognosis of TNBC remains unsatisfactory, with five-year survival rates considerably lower than those of other breast cancer subtypes [14,15]. These persistent clinical challenges underscore the urgent need to identify novel molecular targets and develop more effective therapeutic strategies for TNBC. Recently, cuproptosis has emerged as a promising cell death mechanism with potential therapeutic implications in cancer. Beyond its direct cytotoxic effects on tumor cells, cuproptosis has also been implicated in modulating antitumor immune responses. For instance, a cuproptosis-based immunotherapeutic nanoplatform, consisting of nanoplatelets coated with membranes from PD-1-overexpressing T cells, has been shown to significantly enhance T-cell-mediated antitumor immunity and improve therapeutic efficacy *in vivo*. These findings suggest that targeting cuproptosis may represent a novel therapeutic avenue in cancer treatment and warrant further investigation in TNBC [16].

Cuproptosis was first described in 2022 as a novel form of regulated cell death characterized by copper-dependent cytotoxicity and a distinct molecular mechanism. Unlike other forms of regulated cell death, cuproptosis is closely associated with intracellular copper accumulation and the aggregation of lipoylated mitochondrial proteins [17]. LIPT1 is a pivotal regulator of cuproptosis by controlling the lipoylation status of mitochondrial tricarboxylic acid (TCA) cycle proteins, thereby modulating the availability of copper-binding substrates. Excess intracellular copper binding induces proteotoxic stress and subsequently activates cellular stress responses in tumor cells. Natural products, characterized by their structural diversity, low toxicity, broad biological activities, and multi-target properties, have demonstrated substantial potential for development in tumor-targeted therapy. Moreover, growing evidence suggests that dysregulation of ionic homeostasis extends beyond copper metabolism. Alterations in multiple ions, including sodium, have been implicated in tumor metabolism, cell signaling, and therapeutic responsiveness, highlighting ion homeostasis as an emerging therapeutic target in cancer [18]. Growing evidence indicates that a variety of natural bioactive compounds can effectively trigger cuproptosis in cancer cells by modulating copper homeostasis and disrupting key regulatory pathways of cuproptosis, thereby suppressing tumor cell proliferation, invasion, and metastasis and overcoming therapeutic resistance [19]. Compared with conventional chemotherapeutic agents and synthetic small-molecule compounds, natural product-mediated induction of cuproptosis offers a favorable balance of safety and efficacy, providing novel candidate drugs and therapeutic strategies for the clinical management of malignant tumors [20,21].

A growing body of evidence from both preclinical and clinical studies indicates that extracts derived from traditional Chinese medicine possess considerable therapeutic potential against TNBC. *Trifolium pratense* L. has been widely used in traditional European herbal medicine to treat skin disorders, respiratory diseases, and chronic inflammatory conditions. In modern pharmacological research, *Trifolium pratense* L. has been reported to exhibit a variety of biological activities, including anti-inflammatory, antioxidant, estrogenic, and chemopreventive effects [22,23]. BCA is a major bioactive constituent of *Trifolium pratense* L. and has attracted growing attention for its diverse pharmacological properties. Current research on BCA has primarily focused on its anti-inflammatory, antioxidant, and anticancer activities. Regarding anti-inflammatory effects, BCA has been reported to attenuate inflammatory responses by inhibiting the MAPK signaling pathway [24]. In terms of anticancer activity, BCA has been reported to suppress proliferation and metastatic potential in breast cancer and other malignancies through multiple mechanisms, including induction of apoptosis, cell cycle arrest, inhibition of epithelial–mesenchymal transition (EMT), and modulation of oncogenic signaling pathways such as HER2/ERK/mTOR [25,26]. Building upon these previous findings, our study further demonstrates that BCA exerts pronounced inhibitory effects on TNBC cells.

This study integrates transcriptomic analysis and network pharmacology to investigate the targets and mechanisms underlying BCA-induced cell death in TNBC cells. The aim is to identify key biological pathways and molecular interactions associated with the therapeutic effects of BCA, thereby providing a theoretical basis for the development of novel anti-TNBC agents and more effective therapeutic strategies.

## 2 Methods and Materials

### 2.1 Cell Culture

BT-549 (Cat. CL-0041), MDA-MB-231 (Cat. CL-0150B), and MCF-10A (Cat. CL-0525) cells were obtained from Procell (Procell, Wuhan, China). BT549 and MDA-MB-231 cells were cultured in Roswell Park Memorial Institute (RPMI)-1640 medium (RPMI-1640, Gibco, Cat. C11875, Suzhou, China) supplemented with 10% fetal bovine serum (FBS, Gibco, Cat. 10270-106, Brazil) and 1% penicillin–streptomycin (Solarbio, Cat. P1400, Wuhan, China), MCF-10A cells were cultured in MCF-10A Complete Medium (BDBIO, Cat. C5242-125, Zhejiang, China). All cells were cultured in a 37°C incubator containing 5% CO_2_. All cell lines used in this study were routinely tested for mycoplasma contamination using a PCR-based detection method (Procell, Cat. PB180525, Wuhan, China), and all tests were negative. All cell lines were authenticated by short tandem repeat (STR) profiling and matched to the reference profiles provided by the Cell Bank.

### 2.2 Cell Viability Assay

BT-549 and MDA-MB-231 cells were cultured in RPMI-1640 medium (Gibco, Thermo Fisher Scientific, Cat. No. C11875, Suzhou, China), while MCF-10A cells were maintained in MCF-10A complete medium (BDBIO, Cat. No. C5242-125, Zhejiang, China). Cells were seeded into 96-well plates at a density of 5 × 10^3^ cells per well and then cultured with serum-free medium containing BCA (ChemFaces, Cat. 491-80-5, Wuhan, China) at different concentrations for 24 and 48 h. Specifically, BT-549 cells were treated with 0, 5, 10, 15, 20, 25, 30, 40, and 50 μM BCA, while MDA-MB-231 and MCF-10A cells were treated with 0, 5, 10, 20, 40, 60, 80, 100, and 120 μM BCA. For the cuproptosis inhibition experiment, tetrathiomolybdate (TTM) (Sigma-aldrich, Cat. 323446, St. Louis, MO, USA) was used as a copper chelator to inhibit copper-dependent cell death. Cells were treated with BCA in the presence or absence of TTM under the indicated experimental conditions. The CCK-8 working solution (Biocodons, Cat. ZS03025, Beijing, China) was added to each well at 10 μL per well and incubated for 1 h at 37°C. After incubation, the optical density was measured at 450 nm using a microplate reader (Yomin, UMR-9600T, Hangzhou, China) without reference wavelength correction.

### 2.3 Wound Healing Assay

BT-549 and MDA-MB-231 cells were seeded into six-well plates at a density of 5 × 10^5^ cells per well and cultured until reaching approximately 90% confluence. Horizontal reference lines were drawn on the bottom of the plates before seeding. A vertical scratch was then made across the confluent monolayer using a sterile pipette tip. The wells were then gently rinsed with culture medium to remove detached cells and debris. Subsequently, fresh medium containing BCA at final concentrations of 0 or 5 μM was added, and images were captured at 0 and 24 h using an inverted phase-contrast microscope (SOPTOP-ICX41, 2201283, Zhejiang, China).

### 2.4 Cell Invasion Assay

Matrigel (Corning, Cat. 356234, Shanghai, China) was thawed on ice and diluted with basal medium at a 1:8 ratio before coating the upper chamber. The coated inserts were then incubated to allow gel polymerization. After the Matrigel had fully set, BCA-treated cells were resuspended in serum-free medium and seeded into the upper chamber at a density of 5 × 10^4^ cells per insert, while medium containing 10% FBS (Gibco, Cat. 10270-106) was added to the lower chamber to establish a chemotactic gradient. After 24 h, the non-invading cells on the membrane’s upper surface were gently removed with a cotton swab before fixation and staining. The invaded cells on the lower surface were fixed with cell fixation solution (Biosharp, Cat. 143174, Beijing, China) for an appropriate duration, then stained with crystal violet staining solution (Biosharp, Cat. BL802A, Beijing, China). Images were subsequently captured under an inverted microscope (SOPTOP-ICX41, 2201283, Zhejiang, China), and quantitative analysis was performed using GraphPad Prism 8.0 (GraphPad Software, San Diego, CA, USA). For the wound-healing assay, 5 μM BCA was selected as it produced a reproducible inhibitory effect on wound closure within the observation period. In contrast, the Transwell assays additionally included 1 μM BCA to evaluate the concentration-dependent effects on cell migration and invasion.

### 2.5 RNA-Seq

BT-549 cells were exposed to BCA at its half-maximal inhibitory concentration (IC_50_) or to an equivalent volume of culture medium as a control. Total RNA was extracted using TRIzol reagent (Thermo Fisher Scientific, Cat. 10367621, Carlsbad, CA, USA) according to the manufacturer’s instructions. RNA quality and integrity were assessed before downstream analysis. Further sample processing, including library construction and high-throughput sequencing, was commissioned to Biomarker Technologies Co., Ltd. (BMK, Beijing, China) in accordance with standard Illumina-compatible protocols. Raw sequencing data underwent quality control, and clean reads were used for downstream bioinformatics analysis, including differential gene expression analysis.

### 2.6 Western Blot Analysis

Following drug treatment, RIPA lysis buffer was used to lyse cells on ice. After determining the protein concentration using a BCA (Beyotime, Cat. P0012, Shanghai, China) assay, the collected samples were denatured by boiling at 100°C for 5 min. A total of 50 μg of protein per lane was loaded, and proteins were separated on 10% SDS-PAGE gels (Epizyme, Cat. PG212, Shanghai, China), followed by transfer onto PVDF membranes. The membranes were then blocked with 5% non-fat milk in TBST for 1 h at room temperature to prevent nonspecific binding. Subsequently, the membranes were incubated with primary antibodies at 4°C overnight, then with HRP-conjugated secondary antibodies at room temperature for 1 h. Anti-rabbit IgG, HRP-linked antibody (1:3000, CST, Cat. 7074, Danvers, MA, USA) and anti-mouse IgG, HRP-linked antibody (1:3000, CST, Cat. 7076). The primary antibodies used were as follows: N-cadherin (1:1000, CST, Cat. 13116), E-cadherin (1:1000, CST, Cat. 3915), β-catenin (1:1000, CST, Cat. 8480), Vimentin (1:10,000, Proteintech, Cat. 10366-1-AP, Wuhan, China), β-actin (1:1000, CST, Cat. 4967), CyclinA2 (1:1000, CST, Cat. 67955), CDK1 (1:1000, Abcam, Cat. ab133327, Shanghai, China), CDK2 (1:1000, CST, Cat. 18048), LIPT1 (1:1000, Huabio, Cat. HA722717, Hangzhou, China), FDX1 (1:1000, Abclonal, Cat. A20895, Wuhan, China), Lias (1:1000, Proteintech, Cat. 11577-1-AP), DLAT (1:1000, CST, Cat. 12362), GAPDH (1:5000, Huabio, Cat. HA750022), HSP70 (1:1000, Proteintech, Cat. 10995-1-AP). Protein bands were visualized using the ChemiDoc MP Imaging System (Bio-Rad, Hercules, CA, USA), and band intensities were quantified using Fiji (ImageJ, version 1.53, National Institutes of Health, Bethesda, Maryland, USA). Target protein expression levels were normalized to the corresponding loading control (β-actin).

### 2.7 Identification of DEGs, Functional Enrichment, and Molecular Docking

DEG screening was performed using the DESeq2 package on raw RNA-seq data, after filtering for low-abundance genes (FPKM > 0.5 in control samples and > 0 in treated samples). Differentially expressed genes (DEGs) were defined using thresholds of |log2FC| ≥ 1 and adjusted *p*-value (Benjamini–Hochberg false discovery rate, FDR < 0.05). Using GO enrichment analysis, KEGG pathway analysis, and gene set enrichment analysis (GSEA), we investigated the biological functions and signaling pathways associated with the identified genes. GO and KEGG enrichment analyses were performed using the clusterProfiler R package (R software, version 4.5.0), with gene annotation based on the org.Hs.eg.db database. For GSEA, gene sets were obtained from the Molecular Signatures Database (MSigDB). In addition, the cuproptosis-related gene set was curated by screening the GeneCards database (https://www.genecards.org/) and prior publications. Enrichment results were evaluated using normalized enrichment scores (NES), with statistical significance determined by the adjusted *p*-value (FDR). For molecular docking, the SDF structure of BCA was obtained from PubChem (https://pubchem.ncbi.nlm.nih.gov/), and the LIPT1 (PDB ID: 3A7U) structure was retrieved from the Protein Data Bank (https://www.rcsb.org/). Structures were prepared using AutoDock Tools (version 1.5.7), and docking was performed with AutoDock Vina (version 1.2.3). The optimal binding conformation was selected based on the lowest binding free energy and visualized using PyMOL.

### 2.8 Cell Apoptosis and Cell Cycle Assays

BT-549 and MDA-MB-231 cells were treated with BCA dissolved in DMSO. The final concentrations of BCA were 0, 14, 28, and 56 μM for BT-549 cells and 0, 30, 60, and 90 μM for MDA-MB-231 cells, respectively. The final concentration of DMSO was maintained below 0.1% (v/v). Cells treated with vehicle (0.1% DMSO) served as the control group. All cells were co-incubated with BCA for 24 h. After treatment with BCA, BT-549 and MDA-MB-231 cells were washed with PBS, trypsinized, and collected. The cells were then incubated with the appropriate staining reagents according to the instructions of the cell cycle assay kit (Beyotime, Cat. C1052) and the apoptosis assay kit (Bioprimacy, Cat. PMK0872, Wuhan, China), respectively. For apoptosis analysis, viable cells were defined as Annexin V−/PI−; early apoptotic cells as Annexin V+/PI−; late apoptotic cells as Annexin V+/PI+; and necrotic cells as Annexin V−/PI+. For cell-cycle analysis, the proportions of cells in the G0/G1, S, and G2/M phases were determined from DNA content histograms. Flow cytometry was performed using an Agilent NovoCyte 3005 flow cytometer (Agilent Technologies, Santa Clara, CA, USA). For each sample, at least 10,000 events were acquired. Data acquisition and analysis were performed using NovoExpress software (Agilent Technologies, USA). All experiments were independently repeated at least three times (n = 3).

### 2.9 Measurement of Intracellular ROS, Mitochondrial Membrane Potential, and Intracellular Cu^2^^+^

BT-549 and MDA-MB-231 cells were seeded into six-well plates at a density of 5 × 10^5^ cells per well and incubated for 24 h to allow cell attachment. Subsequently, the cells were treated with culture medium containing the indicated experimental drugs for an additional 24 h. After treatment, the medium was removed, and the cells were washed twice with PBS. For intracellular ROS detection, cells were incubated with 10 μM DCFH-DA (Beyotime, Cat. S0033S) at 37°C for 20 min according to the manufacturer’s instructions. For assessment of mitochondrial membrane potential, cells were stained with 500 μL of JC-1 working solution (Beyotime, Cat. C2006) at 37°C for 20 min according to the manufacturer’s instructions. After washing with JC-1 staining buffer, mitochondrial membrane potential was quantified as the ratio of JC-1 aggregates (red fluorescence) to monomer (green fluorescence). For intracellular Cu^2^^+^ detection, cells were incubated with a 10 μM Cu^2^^+^-specific fluorescent probe (Solarbio, Cat. C8720, Beijing, China) at 37°C for 30 min in the dark. Flow cytometric analysis was performed using a NovoCyte 3005 flow cytometer (Agilent Technologies, Santa Clara, CA, USA). DCF fluorescence was detected in the FITC channel (excitation: 488 nm; emission: 530 ± 30 nm). JC-1 monomers and aggregates were detected in the FITC (excitation: 488 nm; emission: 530 ± 30 nm) and PE (excitation: 488 nm; emission: 585 ± 20 nm) channels, respectively. Intracellular Cu^2^^+^ fluorescence was detected in the PE channel (excitation: 488 nm; emission: 585 ± 20 nm). At least 10,000 events were acquired for each sample. Data acquisition and analysis were performed using NovoExpress software (Agilent Technologies, USA), and fluorescence signals were quantified as the mean fluorescence intensity (MFI). All experiments were independently repeated at least three times (n = 3).

### 2.10 GSH Assay

BT-549 and MDA-MB-231 cells were treated with BCA dissolved in DMSO for 24 h. The final concentrations of BCA were 0, 14, 28, and 56 μM for BT-549 cells and 0, 30, 60, and 90 μM for MDA-MB-231 cells, respectively. Cells treated with 0.1% DMSO served as the vehicle control. Following treatment, intracellular glutathione levels were determined using a GSH and GSSG assay kit (Beyotime, Cat. S0053) according to the manufacturer’s instructions. Briefly, reduced GSH was removed using the GSH-scavenging reagent provided in the kit, thereby enabling selective determination of oxidized glutathione (GSSG). Total glutathione and GSSG levels were measured separately, and reduced GSH levels were calculated accordingly. After incubation for the recommended duration, absorbance was measured at 412 nm using a microplate reader (Yomin, UMR-9600T, Hangzhou, China). Intracellular glutathione levels were quantified based on standard calibration curves provided in the kit. All experiments were independently performed in triplicate (biological replicates, n = 3), and each sample was measured in technical triplicate to ensure reproducibility. The concentrations of GSH and GSSG were calculated using standard calibration curves and expressed as absolute values. No normalization to protein content, cell number, or DNA content was performed.

### 2.11 In Vivo Metastasis Assay

All animal experiments were conducted in accordance with protocols approved by the Animal Ethics Committee of Sichuan University (Permit Number: No. K2024013). The study was designed to evaluate the effect of Biochanin A on breast cancer lung metastasis *in vivo*. Following a one-week acclimation period, six- to eight-week-old female BALB/c nude mice weighing 18–22 g (Chengdu Dashuo Experimental Animal Co., Ltd.) were housed under specific pathogen-free (SPF) conditions with controlled temperature, humidity, and a 12 h light/dark cycle, with *ad libitum* access to food and water. Mice were injected via the tail vein with MDA-MB-231 cells at a concentration of 1 × 10^7^ cells/mL to establish a lung metastasis model. One week after inoculation, a total of 30 mice were randomly assigned to three groups (control, 15 mg/kg BCA, and 30 mg/kg BCA; n = 10 per group) using a random number table. No animals were excluded from the analysis. Biochanin A was administered intraperitoneally once daily for 21 consecutive days. The dose selection was based on previous *in vivo* pharmacological studies. Investigators responsible for outcome assessment were blinded to group allocation during quantification of metastatic nodules and histological analysis. At the end of the treatment period, mice were euthanized, and lungs were harvested, rinsed with PBS to remove residual blood, and macroscopic metastatic nodules were counted. The lung tissues were fixed in 4% paraformaldehyde (Servicebio, G1101-3ML, Wuhan, China) and subsequently processed for hematoxylin and eosin (H&E) staining. Briefly, the fixed lung tissues were dehydrated through a graded ethanol series, cleared with xylene, embedded in paraffin, and sectioned at 4 μm thickness using a microtome. The paraffin sections were deparaffinized, rehydrated, and stained with hematoxylin and eosin using an H&E staining kit (Servicebio, G1076-500ML) according to the manufacturer’s instructions. After staining, the sections were dehydrated, cleared, and mounted with neutral resin. Histopathological changes in lung tissues were observed and photographed using a light microscope (SOPTOP-ICX41, SOPTOP, China). The primary endpoint was the number of pulmonary metastatic nodules, and secondary endpoints included histopathological changes in lung tissues. Statistical analysis was performed using appropriate parametric or non-parametric tests as indicated.

### 2.12 Statistical Analysis

All cell-based experiments were independently performed in triplicate. Statistical analyses were carried out using GraphPad Prism 8.0 (GraphPad, San Diego, CA, USA). Data are expressed as mean ± standard error of the mean (SEM). Comparisons between two groups were evaluated using a *t*-test, while differences among multiple groups were assessed using one-way analysis of variance (ANOVA). Statistical significance was defined as *p* < 0.05. Significance levels were denoted as *p < 0.05, **p < 0.01, ***p < 0.001, and ****p < 0.0001.

## 3 Results

### 3.1 Biochanin A Suppresses Cell Viability and Induces S-Phase Cell Cycle Arrest in TNBC Cells

The chemical structure of BCA is shown in (Fig. 1A). To investigate the effect of BCA on the proliferation of TNBC cells, cell viability was assessed using a CCK-8 assay. BT-549 and MDA-MB-231 cells were treated with increasing concentrations of BCA for 24 and 48 h. BCA significantly inhibited cell viability in both TNBC cell lines in a concentration-dependent manner, and the inhibitory effects were further enhanced after prolonged exposure. The IC_50_ values for BT-549 cells were 26.62 μM and 20.42 μM at 24 and 48 h, respectively. For MDA-MB-231 cells, the corresponding IC_50_ values were 59.64 μM and 36.34 μM, respectively. These findings demonstrated that BCA effectively suppressed TNBC cell proliferation in a dose- and exposure time-dependent manner (Fig. 1B,C).

The estimated IC_50_ values of BCA in MCF-10A cells were 235.8 μM at 24 h and 113.6 μM at 48 h (Fig. 1D). Notably, the 24 h IC_50_ value was extrapolated beyond the highest concentration tested, whereas the 48 h IC_50_ value was within the tested concentration range. These results suggested that MCF-10A cells exhibited lower sensitivity to BCA compared with TNBC cell lines.

BT-549 and MDA-MB-231 cells were subjected to flow cytometry to assess the effects of BCA on cell cycle progression in triple-negative breast cancer cells. Compared with the control group, BCA treatment reduced the proportion of G1-phase cells and increased the proportion of S-phase cells, indicating S-phase cell-cycle arrest in both cell lines. Consistently, Western blot analysis revealed that BCA decreased the expression levels of Cyclin A2, CDK1, and CDK2 (Fig. 1E–G), suggesting that modulation of these cell cycle regulators contributes to BCA-induced S-phase arrest.

**Figure 1:**
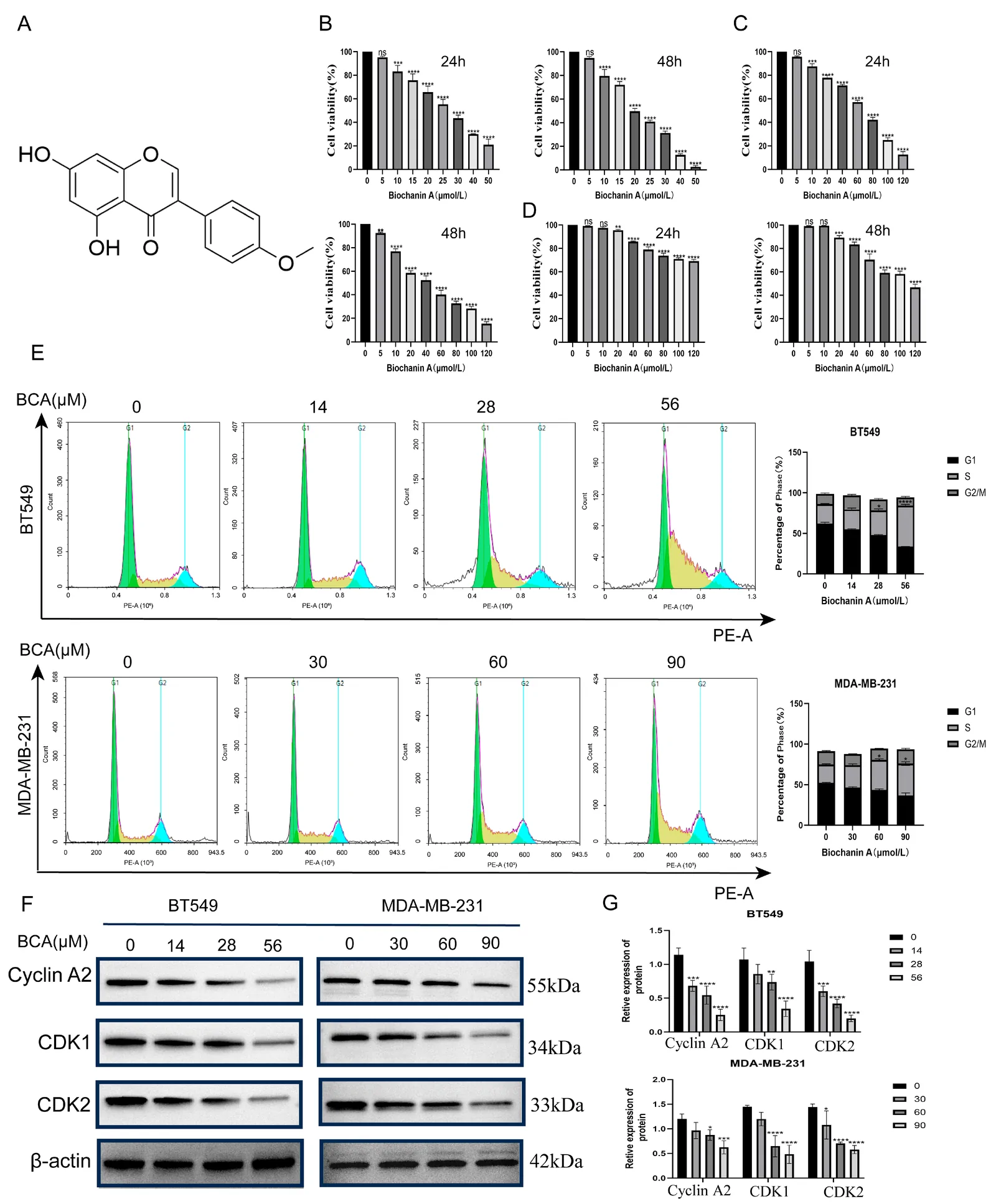
Biochanin A suppresses cell viability and induces S-phase cell cycle arrest in TNBC cells. (**A**) Molecular structure of Biochanin A. (**B**) BT-549 cells were treated with Biochanin A for 24 or 48 h, and cell viability was evaluated using the CCK-8 assay. (**C**) MDA-MB-231 cells were treated with Biochanin A for 24 or 48 h, and cell viability was evaluated using the CCK-8 assay. (**D**) MCF-10A cells were treated with Biochanin A for 24 or 48 h, and cell viability was evaluated using the CCK-8 assay. (**E**) Cell cycle distribution of BT-549 and MDA-MB-231 cells following Biochanin A treatment. (**F**) Western blot analysis of Cyclin A2, CDK1, and CDK2 protein expression in BT-549 and MDA-MB-231 cells treated with BCA. (**G**) Quantification of Western blot protein expression in BT-549 and MDA-MB-231 cells. Statistical significance compared with the control group: ns, not significant; *p < 0.05, **p < 0.01, ***p < 0.001, ****p < 0.0001.

### 3.2 Biochanin A Inhibits the Migration and Invasion of TNBC Cells

We performed wound-healing and Transwell experiments to clarify how BCA treatment affects cell migratory and invasive abilities. Relative to the control group, BCA significantly reduced the migratory ability of both cell lines in a dose-dependent fashion, as reflected by slower wound closure (Fig. 2A,B). Similarly, the Transwell assay showed a reduced number of invading cells after BCA treatment (Fig. 2C,D), demonstrating its strong inhibitory effect on invasion. Given the key role of EMT in metastasis, EMT-related protein expression was analyzed. Western blot results revealed that BCA decreased mesenchymal markers N-cadherin, β-catenin, and Vimentin, while increasing E-cadherin levels (Fig. 2E,F), indicating suppression of EMT. Together, these findings suggest that BCA treatment reduces cell migration and invasion, at least in part by modulating EMT-associated pathways.

**Figure 2:**
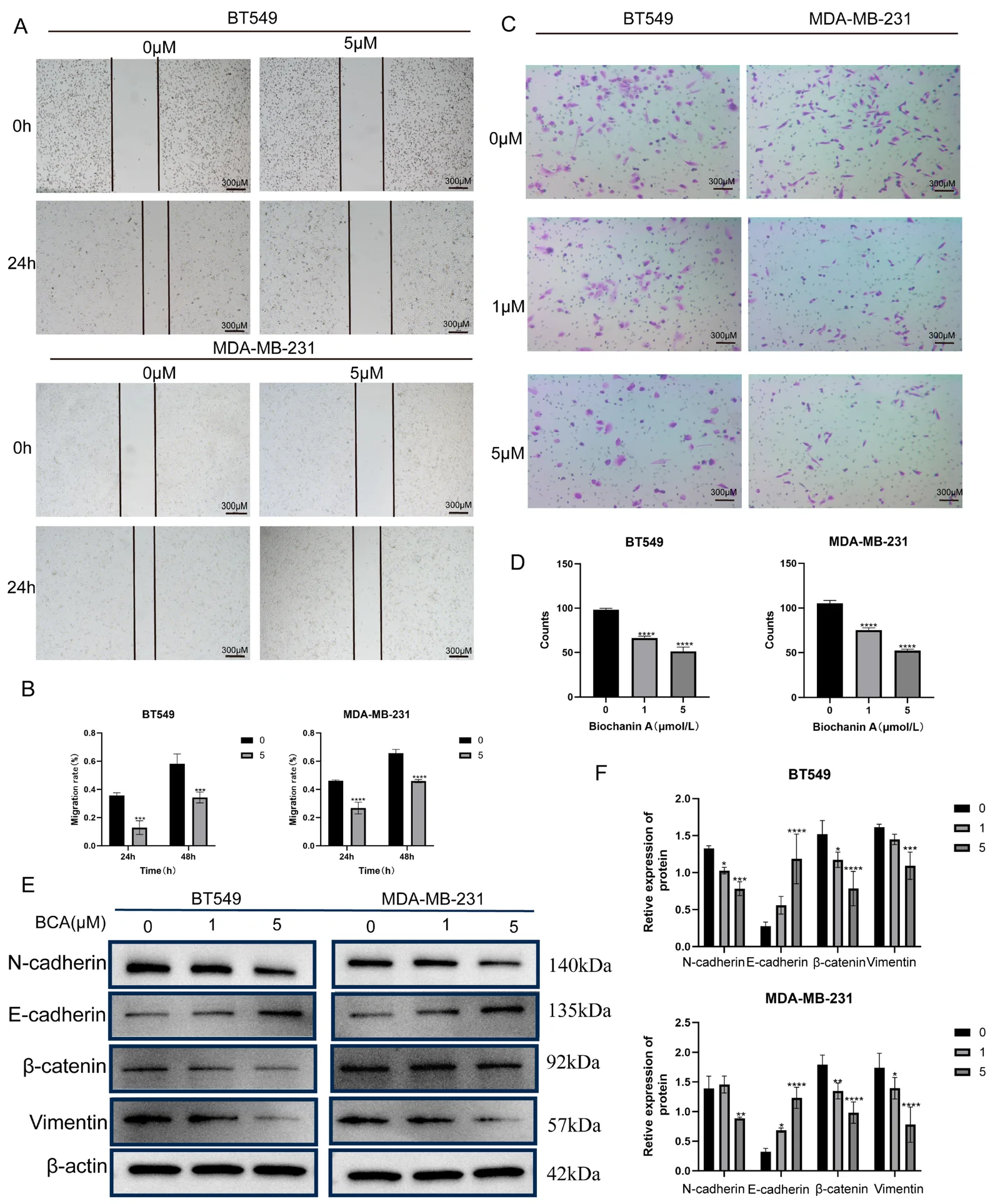
Biochanin A inhibits the migration and invasion of TNBC cells. (**A**) The images of BT-549 and MDA-MB-231 cells treated with Biochanin A for 24 h were captured after scratching (Scale bar: 300 μm), and the effect of Biochanin A on cell gap closure was quantified using ImageJ. (**B**) Quantification of wound healing assay results in BT-549 and MDA-MB-231 cells at 24 h. (**C**) BT-549 and MDA-MB-231 cells were treated with Biochanin A, and Transwell assays were performed. After 24 h of culture, invaded cells were photographed and quantified (Scale bar: 300 μm). (**D**) Quantification of invasion assay results in BT-549 and MDA-MB-231 cells. (**E**) Western blot analysis of N-cadherin, E-cadherin, β-catenin, and Vimentin protein expression in BT-549 and MDA-MB-231 cells treated with BCA. (**F**) Quantification of Western blot protein expression in BT-549 and MDA-MB-231 cells. Statistical significance compared with the control group: *p < 0.05, **p < 0.01, ***p < 0.001, ****p < 0.0001.

### 3.3 Transcriptomic Analysis Identifies Differentially Expressed Genes and Enriched Pathways in BCA-Treated TNBC Cells

To elucidate the potential molecular mechanisms underlying the antitumor effects of BCA, RNA sequencing was performed to compare the transcriptomic profiles of BT-549 cells between the control and BCA-treated groups. The results showed that, compared with the control group, BCA treatment significantly upregulated 868 genes and downregulated 771 genes (Fig. 3A).

KEGG pathway classification results showed that the DEGs were mainly enriched in several functional categories, including cellular processes, environmental information processing, human diseases, metabolism, genetic information processing, and organismal systems (Fig. 3B). Within the cellular process category, endocytosis, cell cycle, and apoptosis were highly represented. In environmental information processing, multiple classical cancer-related signaling pathways were identified, including MAPK and PI3K-Akt signaling pathways. In addition, pathways, transcriptional misregulation, and proteoglycans in cancer were significantly annotated. Overall, these results suggest that BCA may regulate multiple biological processes involved in tumor progression, including cell proliferation, survival, metabolism, and stress response.

GO enrichment analysis results showed that DEGs were mainly enriched in Biological Process (BP) terms, including cellular process, biological regulation, metabolic process, and response to stimulus, suggesting that these genes may participate in a variety of fundamental biological regulatory and stress-response processes. In the Cellular Component (CC) category, DEGs were primarily localized to cellular anatomical entities, intracellular regions, and protein-containing complexes, indicating that the gene products play important roles in cellular structural organization and functional macromolecular complexes. In terms of Molecular Function (MF), DEGs were mainly enriched in binding and catalytic activities and were also associated with transcription regulatory and transporter activities. Overall, the GO analysis suggests that these differentially expressed genes may collectively contribute to disease progression or phenotypic changes by regulating multiple biological processes and molecular functions (Fig. 3C).

Furthermore, transcriptomic analysis suggested that BCA may induce apoptotic signaling in TNBC cells. Annexin V-FITC/PI double staining followed by flow cytometry was performed to assess apoptosis. BCA treatment significantly increased the proportion of late apoptotic cells compared with the control group (Fig. 3D,E), confirming its pro-apoptotic effect in TNBC cells.

**Figure 3:**
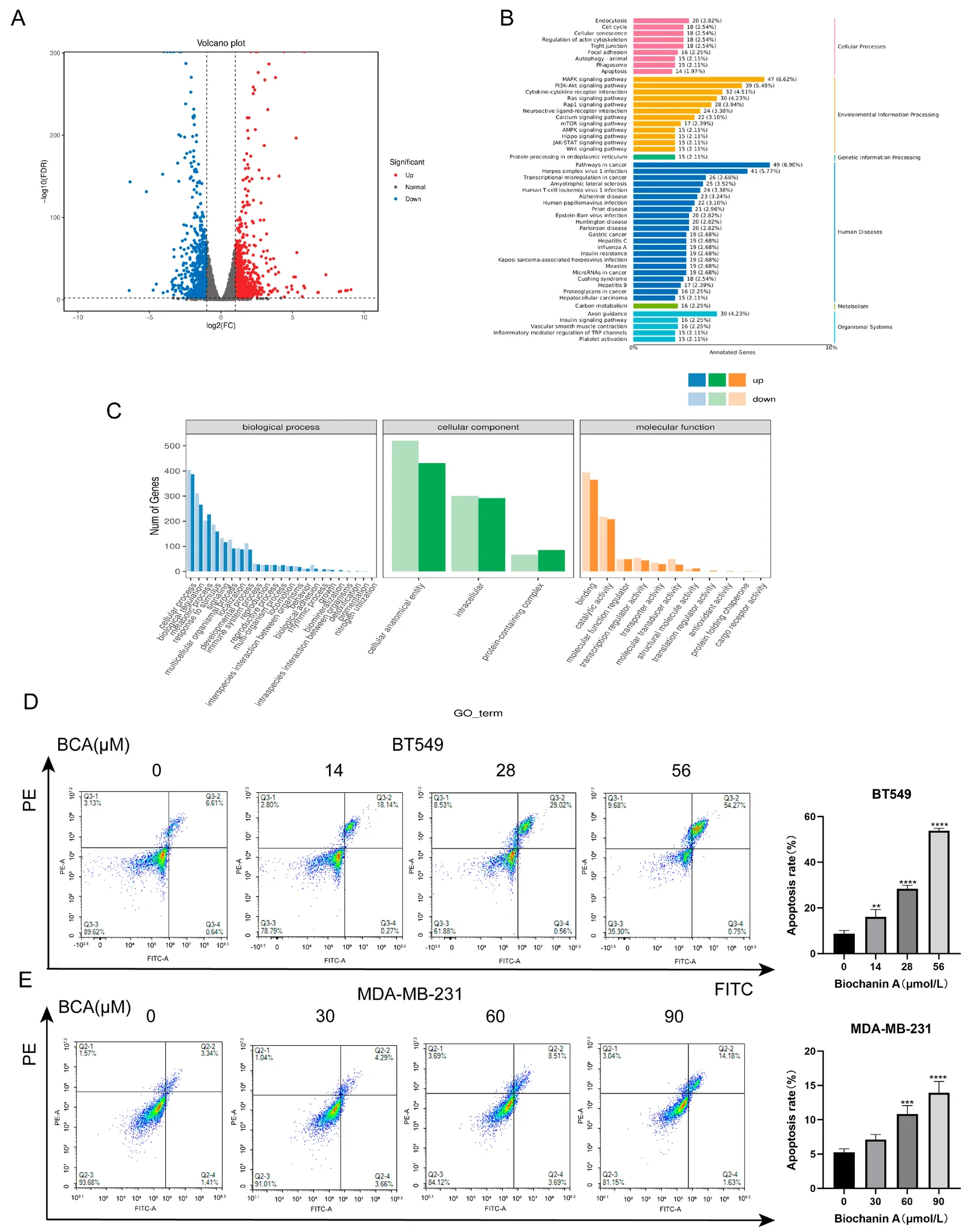
Transcriptomic analysis identifies differentially expressed genes and enriched pathways in BCA-treated TNBC cells. (**A**) A volcano plot was used to visualize genes that exhibited significant changes in expression. (*p* < 0.05, |log2FC| ≥ 1). (**B**) KEGG enrichment analysis screened key pathways contributing to the biological process. (**C**) GO analysis of genes significantly modulated in Biochanin A-treated BT-549 cells. (**D**) Annexin V-FITC/PI apoptosis assay results for BT-549 cells treated with Biochanin A. (**E**) Annexin V-FITC/PI apoptosis assay results for MDA-MB-231 cells treated with Biochanin A. Statistical significance compared with the control group: **p < 0.01, ***p < 0.001, ****p < 0.0001.

### 3.4 Bioinformatic Analysis Identifies LIPT1 as a Potential Candidate Associated with Biochanin A-Induced Cuproptosis

As an essential trace element, copper plays a critical role in mitochondrial function and in maintaining cellular redox homeostasis. Dysregulation of copper homeostasis has been implicated in tumor growth, angiogenesis, and metastatic progression. Cuproptosis is a recently identified form of regulated cell death triggered by intracellular copper accumulation, characterized by mitochondrial protein aggregation and dysfunction of lipoylated enzymes in the TCA cycle. Targeting copper-dependent cell death has therefore emerged as a promising therapeutic strategy for cancer treatment. In this context, natural compounds, including TCM-derived monomers, may induce cuproptosis in cancer cells and provide potential therapeutic opportunities.

To identify potential cuproptosis-related targets of BCA, 1639 differentially expressed genes (DEGs) were identified from RNA sequencing. In parallel, 151 genes associated with cuproptosis were acquired from the GeneCards database. Intersection analysis between the DEGs and the cuproptosis-related gene set identified 12 overlapping candidate genes (Fig. 4A), which were subsequently prioritized based on their relevance to cuproptosis-related signatures (Supplementary Table S1). Among these, LIPT1 showed the highest relevance score and was selected as a candidate gene potentially associated with BCA-induced cuproptosis in TNBC cells. To explore the potential association between BCA and LIPT1, molecular docking analysis was performed. As shown in (Fig. 4B), BCA exhibited a favorable binding affinity toward LIPT1, with a binding energy of −5.9 kcal/mol. Meanwhile, six hydrogen bonds were predicted, suggesting a potential interaction between BCA and LIPT1. Using the Kaplan–Meier Plotter database, we analyzed the expression profile of LIPT1 across different cancer types (Fig. 4C). As shown in (Fig. 4D), LIPT1 exhibited markedly lower expression levels in breast cancer tissues compared with benign breast tissue (*p* = 2.41 × 10^−^^20^). Kaplan–Meier survival estimates indicated that elevated LIPT1 levels predict favorable overall survival in breast cancer patients (Fig. 4E). In GSEA enrichment analysis, NES (Normalized Enrichment Score) represents the normalized gene enrichment score. Pathways with |NES| > 1.5 and *p*-value < 0.05 are generally considered significantly enriched. The GSEA results showed that the cuproptosis pathway had an enrichment score of NES = 2.35 with a *p*-value < 0.001, indicating that the cuproptosis pathway was significantly enriched (Fig. 4F). Finally, Western blot analysis showed that BCA treatment increased LIPT1 protein expression in BT-549 and MDA-MB-231 cells (Fig. 4G,H). These findings suggest that LIPT1 may be involved in the cellular response to BCA treatment and may serve as a potential mediator of BCA-associated cuproptosis in TNBC cells.

**Figure 4:**
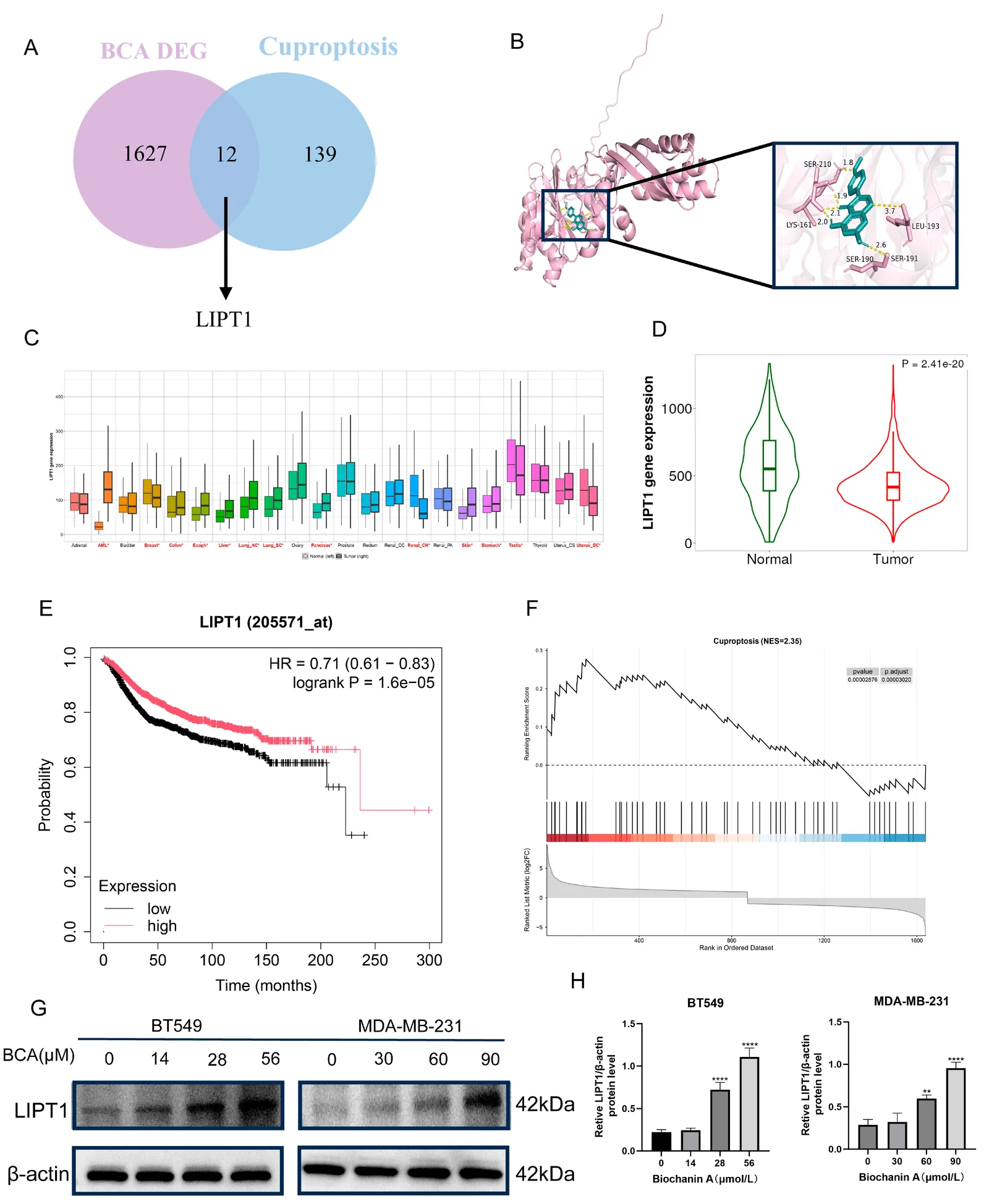
Bioinformatic analysis reveals that Biochanin A targets LIPT1 to regulate cuproptosis. (**A**) Venn diagram of BCA and cuproptosis targets. (**B**) Molecular docking diagram of BCA and LIPT1 protein. (**C**) Using the Kaplan–Meier Plotter database, we analyzed the differential expression of LIPT1 in various cancer types. (**D**) Using the Kaplan–Meier Plotter database, compare LIPT1 expression between breast cancer and normal breast tissue. (**E**) Using the Kaplan–Meier plotter database, the survival curve of breast cancer patients with different LIPT1 expression levels was analyzed. (**F**) The cuproptosis pathway enrichment results of the GSEA enrichment analysis. (**G**) Western blot analysis of LIPT1 protein expression in BT549 and MDA-MB-231cells treated with BCA. (**H**) Quantification of Western blot protein expression in BT-549 and MDA-MB-231 cells. Statistical significance compared with the control group: **p < 0.01, ****p < 0.0001.

### 3.5 Biochanin A Triggers Cuproptosis in TNBC Cells via ROS and Cu^2+^ Accumulation and GSH Depletion

To further determine whether BCA induces cuproptosis in TNBC cells, BT-549 and MDA-MB-231 cells were treated with BCA in the presence or absence of the cuproptosis inhibitor TTM. As shown in (Fig. 5A), BCA treatment significantly reduced cell viability in both cell lines, whereas co-treatment with TTM partially reversed this effect. These findings suggest that inhibition of cuproptosis attenuates BCA-induced cytotoxicity, indicating that BCA-induced cell death is associated with the activation of cuproptosis.

We further assessed several phenotypic changes related to cuproptosis. Intracellular GSH, an important antioxidant and copper-chelating molecule, was significantly reduced following BCA treatment (Fig. 5B), suggesting impaired cellular antioxidant capacity and reduced copper-buffering capacity. In parallel, intracellular ROS levels were markedly increased following BCA exposure (Fig. 5C,D), indicating enhanced oxidative stress that may contribute to mitochondrial dysfunction and cellular damage. In addition, intracellular free Cu^2^^+^ levels were significantly elevated in BT-549 and MDA-MB-231 cells following BCA treatment (Fig. 5E,F), suggesting disruption of copper homeostasis and a permissive environment for cuproptosis.

**Figure 5:**
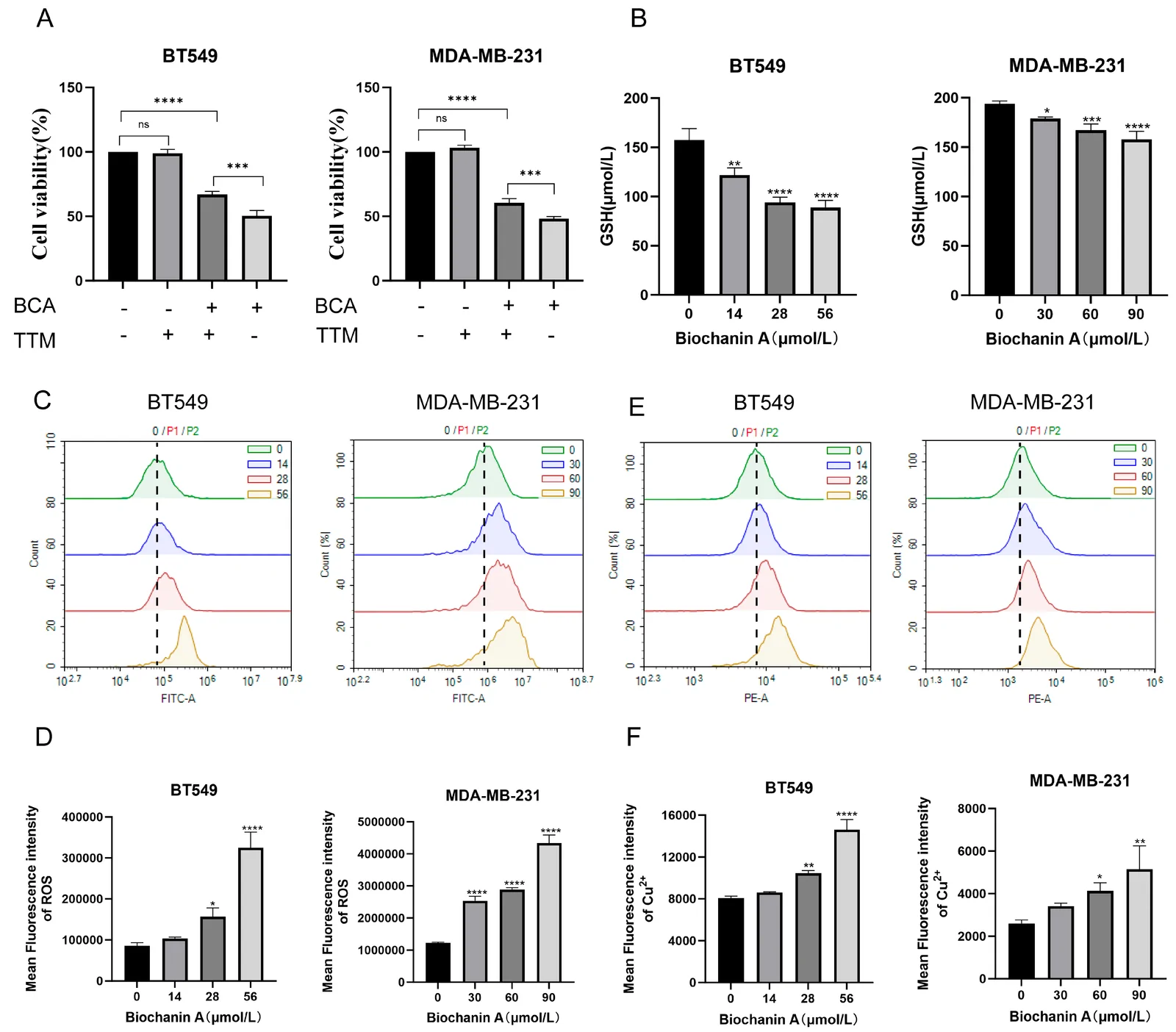
Biochanin A triggers cuproptosis in TNBC cells via ROS, Cu^2+^ accumulation, and GSH depletion. (**A**) Cell viability was assessed by CCK-8 assay in BT-549 and MDA-MB-231 cells pretreated with the cuproptosis inhibitor TTM followed by BCA. (**B**) Intracellular GSH levels in BCA-treated BT-549 and MDA-MB-231 cells. (**C**) Intracellular ROS levels were measured by DCFH-DA fluorescence in BT-549 and MDA-MB-231 cells treated with BCA. (**D**) Quantitative analysis of ROS fluorescence intensity in BT-549 and MDA-MB-231 cells. (**E**) Intracellular Cu^2+^ levels were measured by RBH fluorescence in BT-549 and MDA-MB-231 cells treated with BCA. (**F**) Quantitative analysis of Cu^2^^+^ fluorescence intensity in BT-549 and MDA-MB-231 cells. Statistical significance compared with the control group: ns, not significant; *p < 0.05, **p < 0.01, ***p < 0.001, ****p < 0.0001.

### 3.6 Biochanin A Induces Cuproptosis in TNBC Cells by Reducing Mitochondrial Membrane Potential and Modulating the DLAT, FDX1, and LIAS

Excessive accumulation of copper ions has been reported to impair mitochondrial structure and function by promoting opening of the mitochondrial permeability transition pore, thereby disrupting the proton electrochemical gradient across the inner mitochondrial membrane and decreasing mitochondrial membrane potential. Loss of mitochondrial membrane potential is widely recognized as an indicator of mitochondrial dysfunction and has been associated with the initiation of cuproptosis. Accordingly, mitochondrial membrane potential was assessed in BT-549 and MDA-MB-231 cells following BCA treatment. The results showed that BCA significantly reduced mitochondrial membrane potential in both cell lines, suggesting that BCA may be associated with mitochondrial dysfunction and cuproptosis-related cellular stress (Fig. 6A,B).

During cuproptosis, intracellular copper is reduced from Cu^2^^+^ to Cu^+^ in an FDX1-dependent manner, and Cu^+^ has been reported to bind to lipoylated DLAT preferentially, promoting protein aggregation and inducing mitochondrial proteotoxic stress. This process is accompanied by the activation of cellular stress responses and compensatory upregulation of molecular chaperones such as HSP70, which are involved in protein quality control. In addition, alterations in mitochondrial metabolic state have been associated with changes in key lipoylation-related regulators, including FDX1 and LIAS, reflecting disruptions in mitochondrial metabolic homeostasis. We further examined the expression of cuproptosis-related proteins. We found that BCA induced DLAT oligomerization, upregulated HSP70, and downregulated FDX1 and LIAS (Fig. 6C,D), providing additional evidence that BCA triggers cuproptosis in BT-549 and MDA-MB-231 cells.

**Figure 6:**
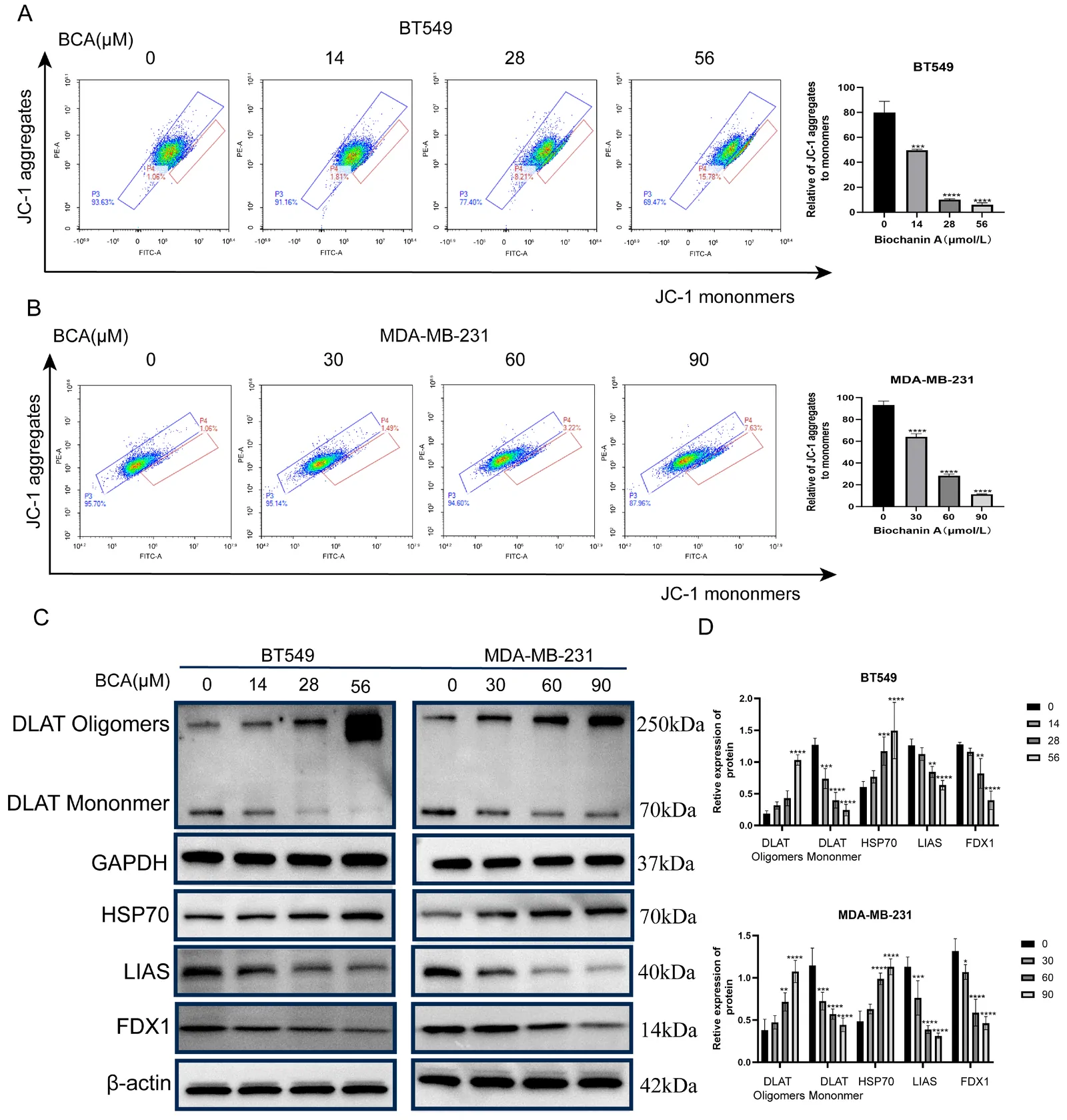
Biochanin A induces cuproptosis in TNBC cells by reducing mitochondrial membrane potential and modulating the expression of DLAT, FDX1, and LIAS. (**A**) JC-1 staining was used to detect alterations in membrane potential after BCA treatment in BT-549 cells. (**B**) JC-1 staining was used to detect alterations in membrane potential after BCA treatment in MDA-MB-231 cells. (**C**) Western blot analysis of DLAT, HSP70, LIAS, and FDX1 protein expression in BT-549 and MDA-MB-231 cells treated with BCA. (**D**) Quantification of Western blot protein expression in BT-549 and MDA-MB-231 cells. Statistical significance compared with the control group: *p < 0.05, **p < 0.01, ***p < 0.001, ****p < 0.0001.

### 3.7 Biochanin A Suppresses In Vivo Metastasis of Human Breast Cancer Cells

A total of 30 female BALB/c nude mice were injected via the tail vein with MDA-MB-231 cells to establish an *in vivo* breast cancer metastasis model. One week after inoculation, the mice were randomly divided into three groups (n = 10 per group) and received intraperitoneal injections of BCA once daily at doses of 0, 15, or 30 mg/kg for 21 consecutive days. After three weeks of continuous treatment, the mice were euthanized (Fig. 7A). The lungs were carefully excised from all animals and rinsed with physiological saline. Metastatic nodules were first assessed by gross examination. Lung tissues were then fixed and processed for H&E staining. Body weight remained stable throughout the treatment period (Fig. 7B).

Macroscopically, the lungs from vehicle-treated mice showed multiple metastatic nodules and foci. In contrast, lungs from mice treated with BCA (15 mg/kg and 30 mg/kg) exhibited markedly fewer metastatic nodules and foci (Fig. 7C,D). Consistently, histopathological analysis by H&E staining showed that, compared with the control group, lungs from BCA–treated mice exhibited reduced, more sparsely distributed metastatic lesions with smaller lesion areas (Fig. 7E).

**Figure 7:**
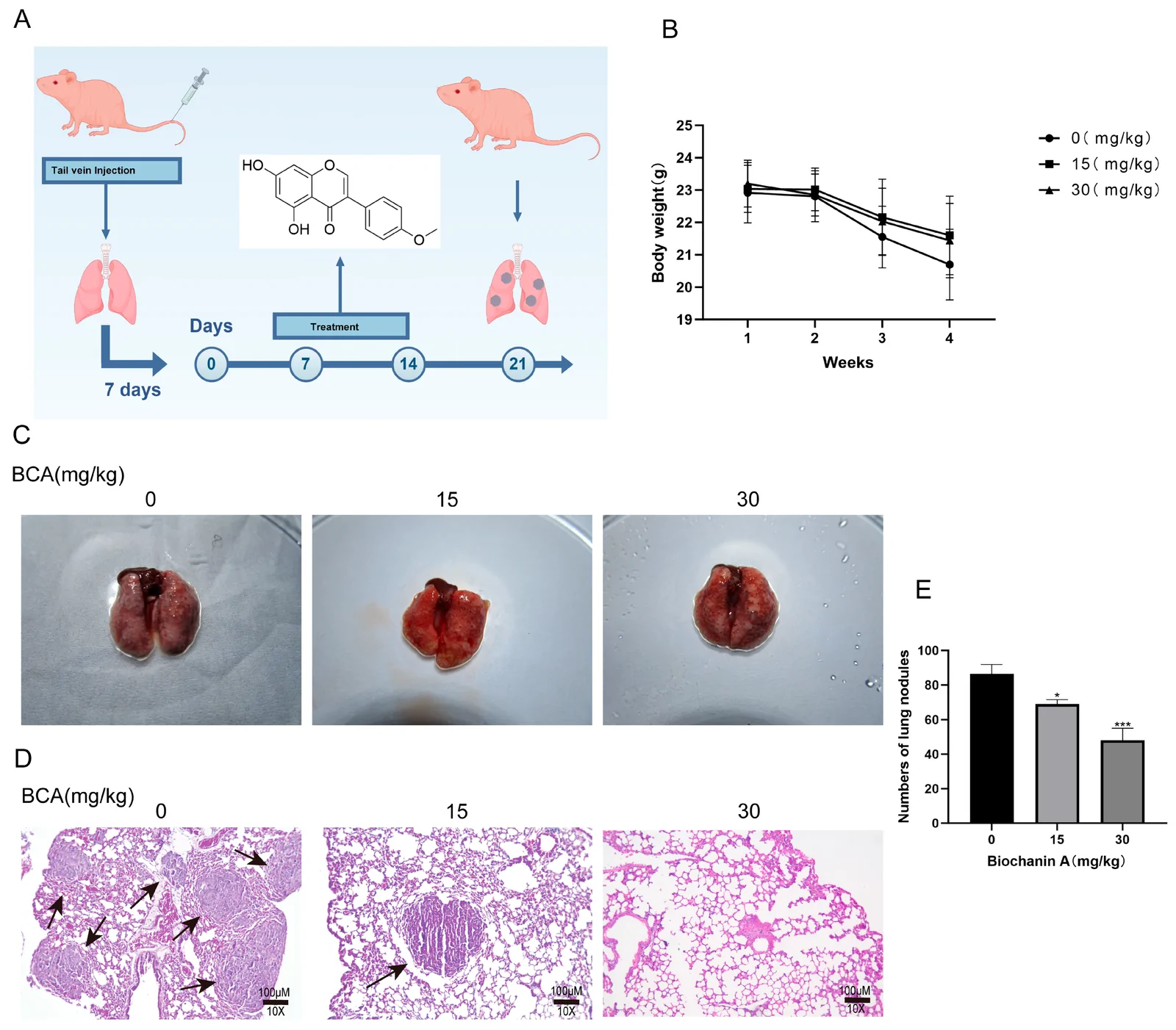
Biochanin A suppresses *in vivo* metastasis of human breast cancer cells. (**A**) Schematic diagram of the *in vivo* metastasis assay model. (**B**) Statistical analysis of body weight changes in mice after Biochanin A treatment. (**C**) After 3 weeks of injecting MDA-MB-231 breast cancer cells into the tail vein, visible metastatic nodules were observed in the lungs of BALB/c-Nude mice. (**D**) Representative H&E-stained images from histological analysis of lung tissue (scale bar: 100 μm), with black arrows indicating metastatic foci. (**E**) The number of metastatic nodules. Statistical significance compared with the control group: *p < 0.05, ***p < 0.001.

## 4 Discussion

The disruption of the tumor microenvironment in TNBC, mediated by interactions among tumor cells, cancer-associated fibroblasts, and infiltrating immune cells, plays a pivotal role in tumor progression, metastasis, and therapeutic resistance. Given the highly aggressive nature of TNBC, its frequent recurrence, and the lack of effective targeted therapies, there remains an urgent need to develop novel, effective treatment strategies [27]. In this context, traditional Chinese medicine and its bioactive natural products have shown promising pharmacological effects across a variety of diseases, offering a potential avenue for treating TNBC.

Previous reports have indicated that *Trifolium pratense* L. has been traditionally used to manage asthma, whooping cough, and cancer, suggesting its historical relevance in anticancer applications. In addition, several studies have noted that *Trifolium pratense* L. has been used in traditional medicine for cancer treatment; however, these applications are largely empirical and lack systematic mechanistic investigation. Recent reviews have further demonstrated that this plant is rich in bioactive constituents, particularly isoflavones, which exhibit antioxidant and anticancer properties and have shown inhibitory effects across various cancer models [28]. Nevertheless, its anticancer efficacy remains predominantly supported by preclinical evidence, with limited clinical validation [29]. BCA is a natural isoflavone compound derived from *Trifolium pratense* L. In the present study, BCA exhibited significant cytotoxic, antiproliferative, antimigratory, and anti-invasive activities and induced apoptosis in tumor cells. Cuproptosis, a newly characterized form of regulated cell death, has emerged as an important mechanism involved in tumor suppression and malignant progression. Owing to their multi-target specificity and relatively low toxicity, natural products are increasingly recognized as crucial inducers of cuproptosis in cancer cells. Therefore, this study investigated the mechanisms by which BCA induces cuproptosis in TNBC cells.

LIPT1 is a mitochondrial lipoyltransferase involved in protein lipoylation, catalyzing the transfer of lipoyl groups from lipoyl-GCSH to mitochondrial metabolic enzyme complexes, thereby contributing to the proper function of TCA cycle-associated enzymes [30]. Emerging evidence indicates that LIPT1 is associated with the regulation of cuproptosis. As an essential component of the lipoylation pathway, LIPT1 contributes to the formation of lipoylated mitochondrial enzymes, including dihydrolipoamide S-acetyltransferase (DLAT), which has been identified as a major copper-binding target among lipoylated proteins [31].

Under conditions of excessive intracellular copper accumulation, copper ions aberrantly interact with LIPT1-mediated lipoylated proteins, leading to their aggregation and disruption of mitochondrial proteostasis [32]. These events are thought to contribute to proteotoxic stress and mitochondrial dysfunction, processes associated with the progression of cuproptosis. In addition, the expression level of LIPT1 directly influences the abundance of lipoylated substrates, thereby modulating cellular sensitivity to copper-induced toxicity [33]. Previous studies have demonstrated that LIPT1 upregulation enhances cellular susceptibility to cuproptosis, whereas its knockdown partially attenuates copper-mediated cytotoxicity. Consequently, LIPT1 is considered a pivotal molecular hub linking mitochondrial metabolic homeostasis to cuproptotic signaling pathways and plays a critical role in regulating cuproptosis [34,35]. In the present study, we observed that BCA markedly upregulated LIPT1 expression. In addition, alterations in several cuproptosis-related proteins, including DLAT, HSP70, LIAS, and FDX1, were observed following exposure to BCA. Specifically, BCA treatment was associated with increased DLAT oligomerization, upregulation of HSP70, and decreased expression of LIAS and FDX1, suggesting mitochondrial stress and disruption of copper-related metabolic homeostasis [36,37]. To further explore the biological context of LIPT1, co-expression and network analyses revealed that LIPT1 is associated with a gene module involved in metabolic and transcriptional regulation (Supplementary Figs. S1–S4), suggesting that it may function within a broader regulatory network rather than as an isolated regulator. Collectively, these findings suggest that LIPT1 may function as a molecular hub linking mitochondrial metabolic homeostasis with cuproptosis-related regulatory networks. However, LIPT1 was identified as a candidate gene through co-expression and metabolic network analyses, suggesting its potential involvement in TNBC-related biological processes, although further causal validation is required. Nevertheless, several limitations of the present study should be acknowledged. Although our findings from transcriptomic analysis, bioinformatic prediction, and experimental validation support the involvement of cuproptosis in BCA-treated TNBC cells, the current evidence does not fully establish that BCA induces cuproptosis specifically through LIPT1. The lack of additional functional validation, including LIPT1 knockdown/overexpression or rescue experiments and copper-chelator-based rescue assays, is a limitation of this study. Future studies incorporating these approaches are warranted to clarify further the causal role of LIPT1 and the contribution of copper-dependent mechanisms to BCA-induced cuproptosis.

Interestingly, transcriptomic analysis revealed significant enrichment of apoptosis-related pathways following BCA treatment, indicating that BCA may trigger complex cellular stress responses. However, cuproptosis is mechanistically distinct from apoptosis; growing evidence suggests that it is primarily driven by mitochondrial metabolic disruption and proteotoxic stress, consistent with the mitochondrial dysfunction and oxidative stress observed in our study. The aggregation of lipoylated mitochondrial proteins, impairment of mitochondrial respiration, and disturbance of redox homeostasis induced by copper overload collectively result in profound mitochondrial dysfunction [38,39]. Importantly, mitochondrial dysfunction is a well-established trigger of intrinsic apoptotic signaling [40]. Therefore, the enrichment of apoptosis-related pathways observed in our transcriptomic data may reflect secondary cellular responses to cuproptosis-induced mitochondrial damage rather than indicating that apoptosis is an independent or dominant mode of cell death induced by BCA. Collectively, these findings suggest that cuproptosis represents the primary mechanism underlying the anticancer effects of BCA in TNBC cells. At the same time, apoptosis-associated signaling alterations are likely downstream consequences of mitochondrial stress and cellular dysfunction.

Recent studies have demonstrated that intracellular GSH depletion may serve as an important upstream event in regulating multiple forms of programmed cell death. For example, a novel GSH-depleting compound was reported to simultaneously induce ferroptosis and cuproptosis in hepatocellular carcinoma [41]. Interestingly, BCA treatment in the present study also resulted in reduced intracellular GSH levels, increased ROS accumulation, and elevated intracellular Cu^2^^+^ concentrations. These findings suggest that disruption of redox homeostasis may represent a critical event contributing to BCA-induced cuproptosis. However, unlike the aforementioned study, our investigation was specifically designed to elucidate the role of cuproptosis in BCA’s antitumor activity. Consistent with this objective, we identified the upregulation of LIPT1 and the perturbation of mitochondrial lipoic acid metabolism as key molecular events associated with BCA-induced cuproptosis in TNBC cells. Collectively, these findings further support the notion that GSH depletion may facilitate copper-dependent cell death and highlight cuproptosis as an important mechanism underlying the anticancer activity of BCA. Future studies may investigate whether natural small molecules, such as BCA, can coordinate the modulation of distinct cell death programs in specific cellular contexts. Elucidating potential crosstalk and cooperative regulation among distinct death pathways may yield new insights into the development of multi-target therapeutic strategies and deepen our understanding of how small molecules achieve potent anticancer activity through integrated cell death networks.

In addition to its role in regulating cuproptosis, BCA, a naturally occurring isoflavone, may also exert antitumor effects through epigenetic and immunoregulatory mechanisms in TNBC. Accumulating evidence has demonstrated that isoflavones possess epigenetic regulatory properties, including modulation of DNA methylation and histone modifications, which may contribute to their antitumor activity across multiple cancer types [42]. Meanwhile, epigenetic alterations have increasingly been recognized as key regulators of tumor immune microenvironment remodeling and therapeutic response in TNBC [43]. Therefore, the epigenetic regulatory activity of BCA may represent an additional mechanism that complements its cuproptosis-associated effects. Although the present study primarily focused on cuproptosis-related molecular events and the potential involvement of LIPT1-associated mitochondrial metabolic regulation, the findings suggest that BCA may exert multifaceted antitumor effects through coordinated modulation of regulated cell death pathways, mitochondrial metabolism, and epigenetic networks. Further studies are warranted to elucidate the broader regulatory roles of BCA in epigenetic remodeling and tumor immunity, as well as their potential interplay with cuproptosis.

Metastatic dissemination of breast cancer cells, particularly to the lungs, requires substantial mitochondrial adaptability, maintenance of redox homeostasis, and increased resistance to oxidative stress [38]. In the present study, an experimental lung metastasis model was established to evaluate the anti-metastatic activity of BCA. Metastasis is a complex, multistep process that relies on high metabolic plasticity and sustained mitochondrial activity to support tumor cell survival and colonization at distant sites [44,45]. Emerging studies indicate that aberrant mitochondrial function and disrupted redox balance play critical roles in restricting metastatic potential [46,47]. Given that cuproptosis is characterized by mitochondrial metabolic dysfunction and proteotoxic stress, it has emerged as a potential mechanism that may contribute to the suppression of tumor metastasis. Given that cuproptosis is characterized by mitochondrial metabolic dysfunction and proteotoxic stress, it has emerged as a potential mechanism that may contribute to the suppression of tumor metastasis. Consistent with our *in vitro* findings, BCA treatment was associated with increased protein lipoylation, increased oxidative stress, and impaired mitochondrial function. These molecular alterations coincided with reduced migratory and invasive capacities and inhibition of EMT in TNBC cells. Moreover, BCA markedly suppressed lung metastasis *in vivo*. However, because cuproptosis-related molecular markers were not directly evaluated in metastatic lung tissues, a causal relationship between cuproptosis induction and metastasis inhibition could not be established in the present study. Future investigations incorporating mechanistic validation in animal tissues will be necessary to determine whether activation of cuproptosis directly contributes to the anti-metastatic effects of BCA.

Nevertheless, several limitations of the present study should be acknowledged. Although the TTM rescue experiment provides pharmacological support for the involvement of cuproptosis in the anticancer effects of BCA, the mechanistic evidence remains incomplete. First, although our transcriptomic, molecular docking, and protein expression analyses collectively suggest that LIPT1 may be involved in BCA-induced cuproptosis, its functional role has not been directly validated using genetic approaches such as knockdown or overexpression. Furthermore, rescue experiments using genetic manipulation of LIPT1 were not performed to determine whether modulating LIPT1 directly affects the sensitivity of TNBC cells to BCA-induced cuproptosis. Second, although BCA significantly inhibited lung metastasis *in vivo*, cuproptosis-related molecular markers were not assessed in metastatic lung tissues, thereby limiting direct mechanistic validation. Therefore, although our findings support the involvement of cuproptosis in the anticancer activity of BCA, the causal contribution of LIPT1 to BCA-induced cuproptosis and its relationship to the suppression of lung metastasis remain to be further established. Finally, the pharmacokinetic properties and long-term safety profile of BCA were not evaluated in the present study. Future studies integrating genetic manipulation of key cuproptosis regulators, particularly LIPT1, together with complementary rescue experiments and mechanistic validation *in vivo*, will provide a more comprehensive understanding of the molecular basis of BCA-induced cuproptosis. In addition, although no significant body weight loss was observed during treatment, comprehensive safety evaluations, including hematological, biochemical, hepatic, renal, and histopathological assessments, were not performed. Therefore, the safety profile of BCA remains to be systematically evaluated in future studies.

## 5 Conclusions

In summary, BCA, a major bioactive constituent of *Trifolium pratense* L., exhibits significant antitumor activity against triple-negative breast cancer in both *in vitro* and *in vivo* models. The present study suggests that the anticancer effects of BCA are associated with the induction of cuproptosis, accompanied by disruption of copper homeostasis, oxidative stress, mitochondrial dysfunction, and alterations in cuproptosis-related proteins. Bioinformatic and experimental analyses further indicate that LIPT1 may mediate this process; however, its precise functional role requires further investigation. Collectively, these findings provide new insights into the potential mechanisms underlying the anticancer activity of BCA and support further investigation of BCA as a promising natural compound for the treatment of triple-negative breast cancer.

## Data Availability

The raw transcriptome sequencing data generated in this study have been deposited in the NCBI Sequence Read Archive (SRA) under accession number PRJNA1429079. The experimental dataset generated in this study is available from the corresponding author upon reasonable request.

## Supplementary Materials

The supplementary material is available online at https://www.techscience.com/doi/10.32604/or.2026.084760/s1.

## Abbreviations

BCA	Biochanin A
BP	Biological Process
CC	Cellular Component
DEG	Differentially expressed gene
DLAT	Dihydrolipoamide S-acetyltransferase
EMT	Epithelial–mesenchymal transition
ER	Estrogen receptor
FDX1	Ferredoxin 1
GSEA	Gene set enrichment analysis
H&E	Hematoxylin and eosin
HER2	Human epidermal growth factor receptor 2
HSP70	Heatshockprotein 70
LIAS	Lipoic Acid Synthetase
LIPT1	Lipoyltransferase 1
MAPK	Mitogen-activated protein kinase
MF	Molecular Function
MsigDB	Molecular Signatures Database
NES	Normalized enrichment scores
PR	Progesterone receptor
SPF	Specific pathogen-free
TCA	Tricarboxylic acid
TMB	Tumor mutational burden
TNBC	Triple-negative breast cancer
TTM	Tetrathiomolybdate
