# Supplementary material for: Biochanin A Induces Cuproptosis and Inhibits Lung Metastasis in triple-negative Breast Cancer
Source: Oncol Res. 2026 Oct 28;34(11):26. doi: 10.32604/or.2026.084760 (PMC13732377; doi:10.32604/or.2026.084760)
Supplement: Supplementary file 1 [file OncolRes-34-84760-s001.zip › TSP_OR_84760-s001.docx]

**Supplementary Table S1**

| **Rank** | **Gene Symbol** | **Relevance score** |
| --- | --- | --- |
| 1 | LIPT1 | 2.083852 |
| 2 | PPP1R15A | 1.442786217 |
| 3 | C5AR1 | 1.42006731 |
| 4 | CGRRF1 | 1.349241734 |
| 5 | PCSK9 | 1.22013557 |
| 6 | TRIM16 | 1.166563988 |
| 7 | UNC5B | 1.166563988 |
| 8 | SHCBP1 | 1.166563988 |
| 9 | SQSTM1 | 0.199931726 |
| 10 | CCNA2 | 0.199931726 |
| 11 | DDIT3 | 0.199931726 |
| 12 | ATF3 | 0.199931726 |

**Supplementary Figure S1**

**Figure S1.** Correlation analysis between LIPT1 and co-expressed genes in breast cancer (BRCA). (A–B) Pearson correlation analysis was performed to evaluate the relationship between LIPT1 expression and the expression levels of REV1 (A) and RBM43 (B) in BRCA samples. The results showed positive correlations between LIPT1 and REV1 (Pearson correlation coefficient = 0.57) and between LIPT1 and RBM43 (Pearson correlation coefficient = 0.53), suggesting that LIPT1 may be associated with specific gene expression networks in breast cancer.

**Supplementary Figure S2**

**Figure S2.** Correlation analysis between LIPT1 and co-expressed genes in breast cancer (BRCA). (A–B) Pearson correlation analysis was performed to evaluate the relationship between LIPT1 expression and the expression levels of UNC50 (A) and ZNF638 (B) in BRCA samples. The results showed positive correlations between LIPT1 and UNC50 (Pearson correlation coefficient = 0.51) and between LIPT1 and ZNF638 (Pearson correlation coefficient = 0.51), suggesting that LIPT1 may be associated with specific gene expression networks in breast cancer.

**Supplementary Figure S3**

**Figure S3.** Correlation analysis between LIPT1 and co-expressed genes in breast cancer (BRCA). (A–B) Pearson correlation analysis was performed to evaluate the relationship between LIPT1 expression and the expression levels of ZNF2 (A) and C2orf64 (B) in BRCA samples. The results showed positive correlations between LIPT1 and ZNF2 (Pearson correlation coefficient = 0.51) and between LIPT1 and C2orf64 (Pearson correlation coefficient = 0.5), suggesting that LIPT1 may be associated with specific gene expression networks in breast cancer.

**Supplementary Figure S4**

**Figure S4.** LIPT1-centered co-expression network analysis in breast cancer (BRCA). The network was generated using the UALCAN database (https://ualcan.path.uab.edu/index.html) based on gene expression correlation analysis from BRCA samples. LIPT1 was identified as a central gene associated with multiple co-expressed genes, including NME family members, LIPT2, ACSL4, and other metabolism-related genes. This analysis suggests that LIPT1 may be involved in broader breast cancer-related molecular networks
